# An in vitro evaluation of the efficacy of a novel iontophoresis fluoride tray on remineralization

**DOI:** 10.4317/jced.51376

**Published:** 2014-10-01

**Authors:** Gonca Girenes, Tezer Ulusu

**Affiliations:** 1 Department of Pediatric Dentistry, Faculty of Dentistry, Gazi University, Ankara, Turkey

## Abstract

Objectives: The aim of this study is to determine the effects on remineralization of a novel iontophoresis device called ‘Fluorinex’, conventional (acidulated phosphat fluoride) APF gel treatment, and conventional ionthophoresis device comparatively by laser fluorescence measurements. Study Design: Artificial incipient carious lesions were created on immature, 60 intact premolar and molar teeth with no defects. The specimens were randomly allocated to four groups. In the first group 1.23% APF gel was applied to specimens by conventional method for 4 minutes and in the second group 2% (sodium fluoride) NaF solution applied by conventional iontophoresis device for 4 minutes. In Fluorinex group specimens were pretreated with (copper chloride) CuCl2 for 1 minute and then treated for 4 minutes with 1.23% APF gel in a Fluoritray. Control group was placed in distilled water for 4 minutes. After these applications all specimens were included to a pH cycling. DIAGNOdent pen measurement were obtained in three different time intervals; after incipient carious lesions, after fluoride treatments and after pH cycling. Specimens were studied by SEM(scanning electron microscopy) after artificial caries lesions and fluoride treatments. Results: Alterations on DIAGNOdent pen measurements before and after treatment, the Fluorinex group was statistically different from conventional APF gel (p=0.011), conventional NaF iontophoresis (p<0.001) and control group (p<0.001). As the DIAGNOdent pen measurements before treatment and after pH cycling were compared, differences were statistically significant in Fluorinex and conventional APF gel groups (p<0.001). Conclusions: The results of this in vitro study has shown that fluoride application by Fluorinex was superior to the conventional APF gel application and NaF iontophoresis on incipient carious lesions.

** Key words:**Fluoride, iontophoresis, remineralization.

## Introduction

Recent studies have focused on non-invasive treatment strategies for early caries lesions (1). The effectiveness of topical fluoride as a cariostatic agent has been well established and professional topical fluoride applications are commonly used to arrest the progression of active caries (2). The principle mechanism of topical fluoride applications is to form calcium fluoride [CaF2] on the enamel surface. The CaF2 on the tooth surface can act as fluoride reservoir to promote remineralization of enamel. However, CaF2 on the enamel surface is easily dissolved within 24 h. This problem could be overcome by penetrating fluoride ions into lesions in a more effective manner. Fluoride iontophoresis [FI] could be an alternative approach to achieve this goal, which is proposed to afford a more effective use of fluoride for caries prevention. Iontophoresis is a method of electrically transporting ionic particles into hard or soft tissue (3). Scientific exploration of the technique started a century ago and with the progress made since 1970, the first commercial devices are now available in the market. The symmetry of iontophoresis, that is, the fact that molecules can not only be delivered into the body but also be extracted, makes the technique equally attractive for the clinical monitoring of drugs and biomarkers (4). This technique is widely used to promote therapeutic effects of drugs in medicine, exactly in dermatology, physiotherapy, and cardiology. As well as it was introduced into dentistry to be used in dentin hypersensitivity and still used in many areas, such as, topical anesthesia (5), temporamandibuler joint disorders (6), endodontics (7), cavity liners and adesive system applications (8,9), and remineralization of early caries lesions (2,3).

Most studies assessing FI were based on decreasing the dentine hypersensitivity. As well as recent studies showed that FI have efficiency on remineralization of incipient caries lesions (2,3) and manufacturing companies have suggested that FI has an effect not only on dentine hypersensitivity but also on caries prevention. However, there was insufficient scientific evidence to support the superiority of FI over conventional fluoride application [CFA] (3).

The iontophoretic application of fluoride into enamel by electrolysis of an aqueous fluoride aims to produce negative fluoride ions that replace the hydroxyl group in the enamel. The amount of fluoride ions delivered is directly proportional to the quantity of energy, which is related to time and current. Due to the polarization produced by the body, a high resistance to fluoride ion flow was generated, thus iontophoretic devices failed to produce high fluoride precipitation (10).

Fluorinex® treatment is a novel procedure for active topical fluoridation which includes; a pretreatment wash with CuCl2 solution, aims at enhancing the electric conductivity of the teeth. The elimination of the polarization effect by applying low direct electrical current through the fluoride gel to teeth via the Fluoritray®. This process facilitates the “active” substitution of the hydroxyl group with the fluoride ions.

The Fluorinex process is similar to that used in electro-palating and called electro-deposition. The part to be palated [tooth] is connected to the cathode of the circuit. The cathode and the anode are immersed in an electrolyte [fluoride gel] containing one or more dissolved metal salts as well as other ions that permit the flow of electricity. When a direct current is supplied to the cathode the ions in the electrolyte solution lose their charge and plate out on the cathode and mainly on the tooth. This device is different from the others, because electric current is passing not through the patient’s body, just through the teeth. The investigators claimed that by this way fluoride ions are penetrating deeper layers of the enamel so its efficiency is getting much and long lasting. On-going *in-vivo* clinical trials have shown that Fluorinex ensures a much higher rate of fuoride uptake than existing commercial gel treatment presently in use (11,12).

The purpose of the present study was to determine the effects on remineralization of ‘Fluorinex’, conventional APF gel treatment, and conventional ionthophoresis device comparatively by laser fluorescence measurements and to observe the changes in enamel occured by different treatment techniques by SEM.

## Material and Methods

Sixty extracted caries-free immature human third molars and premolars were collected and stored at 0.1% tymol solution. Teeth with hypoplastic areas, cracks, or gross irregularities of the enamel structure were excluded from the study. The criteria for tooth selection dictated no pre-treatment with a chemical agent such as alcohol, formalin, hydrogen peroxide, and so forth. Soft tissue remnants and debris were removed from the teeth, following which they were cleaned with a fluoride-free pumice and rubber cup. The roots were removed at the cementoenamel junction and each crown surfaces of all teeth was painted with two coats of acid-resistant varnish, leaving an exposed window of enamel [approximately 4.0 X 4.0 mm] on the middle third of the buccal surface, so that most of the crown was covered by acid resistant varnish, and only the exposed enamel would be attacked by acid.

- Lesion Formation

All of the teeth used in the study were immersed in the demineralizing solution for 96 hours at 37°C to produce artificial incipient caries lesions. Each crown was immersed individually and demineralizing solution was changed daily when pH of the solution checking every day. The demineralizing solution was containing 100 mmol/L lactic acid, 3 mmol/L CaCl2, 1.8 mmol/L KH2PO4, 1% carboxymethyl cellulose with pH 4.0. After 96 hours artificial incipient lesions were created and samples were rinsed with distilled water before DIAGNOdent pen measurements. All created lesions were measured and obtaining scores were about 13-20 which were explained in the instructions for use by Lussi *et al.* (13) as ‘enamel lesions’. By DIAGNOdent pen measurements in all samples, producing artifical enamel lesions were standardized and checked.

- Treatment procedures

To apply fuoride treatments, the specimens were divided randomly into four groups.

Group 1, conventional gel group [APF gel]: 15 specimes were dried with air syringe and placed in 1.23% APF gel [Sultan, New Jersey, ABD. pH 3.5] for 4 minutes.

Group 2, conventional iontophoresis group [NaF solution]: 15 demineralized specimens were dried and 2% NaF solution was applied with 200 microampers for 4 minutes with a fluoride iontophoresis device [Mikromedikal Ltd, Ankara, Turkey]. This device has two poles; the positive anode and the negative cathode was placed in the fluoride source applied to the enamel. This condition allowed current to pass through the fluoride source onto enamel surface.

Group 3, Fluorinex group [APF gel]: In the first step, specimens in this group were placed in 1% CuCl2 solution contained in the Fluorinex set, in a silicon tray for 1 minute according to manufacturer’ s intructions. Then rinsed with distilled water, 1.23% APF gel was applied to the specimens with Fluorinex device [Fluorinex Ltd, Nazareth, Israel] for 4 minutes.

Group 4, control group [distilled water]: 15 specimens were placed in distilled water for 4 minutes without any fluoride treatment.

- pH cycling

The daily procedure of pH cycling included a demineralization period of 6 hours and remineralization of 17 hours. The solutions were prepared as ten Cate and Duijsters (14) at Department of Pharmaceutic Chemistry, Faculty of Pharmacy of Gazi University. Each crown was immersed individually in demineralizaton solution containing 1.5 mM CaCl2, 0.9 mM KH2PO4, 50 Mm acetic acid with pH 4.8 for 6 hours at 37 0C. Specimens were then removed from demineralization solution, rinsed with distilled water and immersed individually in remineralization solution containing 1,5 mM CaCl2, 0,9 mM KH2PO4, 130 mM KCl and 20 mM Hepes with pH 7.0 for 17 hours at 37 0C to stimulate the remineralization stage of caries process. This cycling system was repeated daily for 14 days.

- DIAGNOdent pen measurements

At three different time intervals DIAGNOdent pen readings were obtained; after demineralizing, after fluoride treatments and after pH cycling procedure. The same DIAGNOdent pen was used throughout the study, and one examiner performed the measurements. Before each session, the instrument was calibrated against the ceramic standard supplied by the manufacturer and then calibrated for each subject by measuring a sound area of the buccal surface. The surface was dried with compressed air for 5 seconds before measurement and the same probe tip 3 was used. At each buccal surface two sides were determined, these were the upper corner points of the square at mesial and distal. By this way, if scores will be different between mesial and distal points in the same sample was investigated. Each point was scanned two times with the pen, and the highest value from the two readings was registered. For analysis of the reproducibility of the DIAGNOdent pen measurements, the same examiner undertook the DIAGNOdent examinations in duplicate with an interval of 1 week after the last measurements.

- SEM analysis

Five samples for each group were examined by scanning electron microscopy. One half of the same crown was analized after demineralization, and the other half was treated with different fluoride applications and insert in pH cycling. SEM examinations were done after fluoride therapies and pH cycling.

- Statistical analysis

Data analysis was performed by using Statistical Package for Social Sciences, [SPSS, Vers. 11.5, SPSS Inc. Chicago, Illinois, USA] Means and standard deviations for each group were used for descriptive statistics. Repeated measurements of ANOVA followed by a Greenhouse-Geisser test compared the differences between the treatment groups. The level of significance chosen for tests was *p*<0.05. The differences in the DIAGNOdent pen values that obtained from the corelation between time and groups was analyzed by Bonferroni one way variance analysis followed by post-hoc Tukey HSD test. The level of significance for Bonferroni correction was *p*<0.0167. The one-way ANOVA test was used for comparing the before treatment values among the groups at a significance level of 95%.

Intra examiner reproducibilty were evaluated by intra-class correlation coefficient [ICC] and the limits of aggrements were between 95% confidence intervals.

## Results

At mesial sides intra examiner reproducibilty was 100% and at distal sides the ICC values were revealed an excellent intraexaminer reproducibility again; ICC was between 0.9979 and 0.9993. At all sides, the reproducibility between DIAGNOdent pen measurements obtained after pH cycling and one week later was 99.96%. No significant differences were found [*p*>0.05] regarding the before treatment DIAGNOdent pen values among the groups.

Before testing the significant differences, the interaction terms were analysed and the interaction results indicated that only group and time was found significant ( Table 1).

**Table 1:**
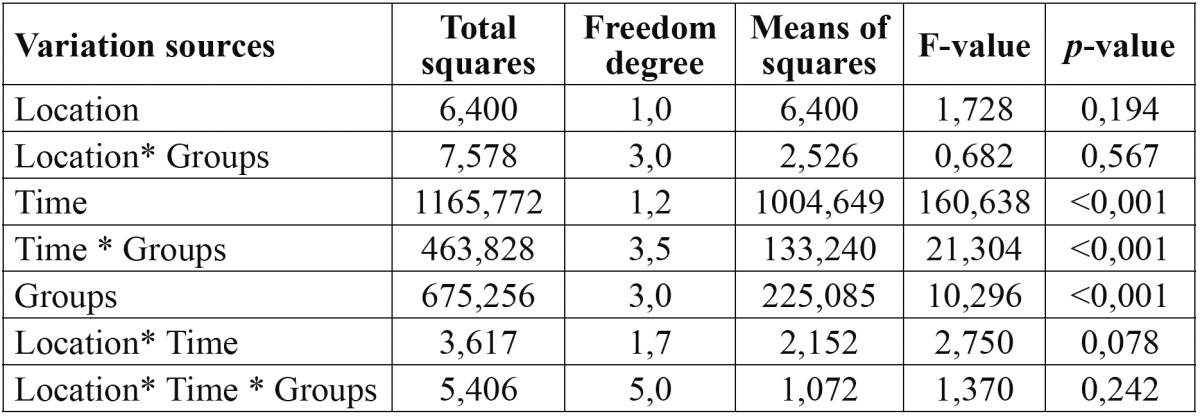
The results of interaction analysis on DIAGNOdent pen measurements.

The avarage of DIAGNOdent measurements of all groups at different time intervals are shown in  Table 2. No significant differences were found [*p*=0.256] regarding the before treatment DIAGNOdent values among the groups.

**Table 2:**
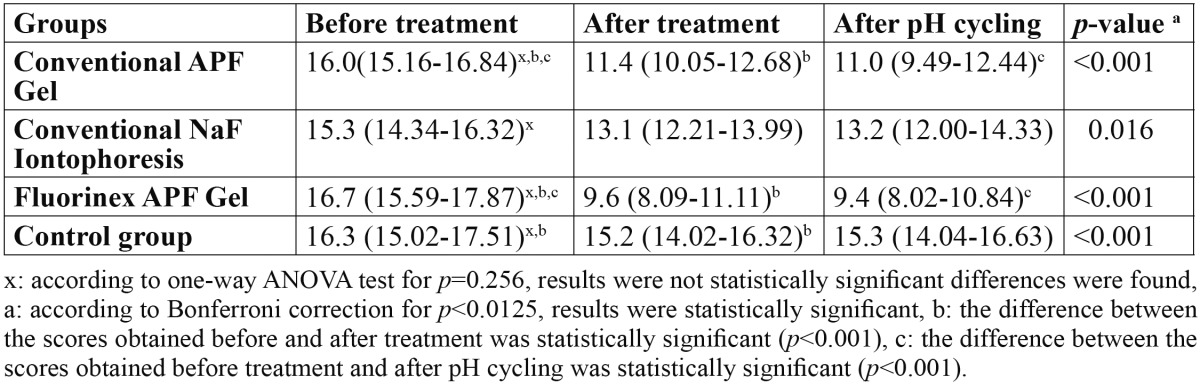
The DIAGNOdent pen measurement values at different times according to the groups.

In conventional APF gel group and Fluorinex group, statistically different values were obtained at the initial enamel lesions before and after the treatment [*p*<0.001]. Conventional NaF group showed no statistically significant difference at DIAGNOdent pen measurement before and after the fluoride treatment [*p*>0.001]. When control group showed statistically significant difference among the values obtained after demineralization and after distilled water application [*p*<0.001], it showed no statisitically difference among the measurements done after demineralization and pH cycling [*p*=0.031]. When the DIAGNOdent values obtained after demineralization and after pH cycling were compared, only in the APF gel and Fluorinex group there was a statistically significant decrease at values [*p*<0.001].

When pretreatment and post-treatment scores of DIAGNOdent pen compared between different groups, the differences between Fluorinex gel group and conventional APF gel group [*p*=0.011], Fluorinex gel and conventional NaF iontophoresis group [*p*<0.001], Fluorinex gel and control group [*p*<0.001] were statistically significant. At the same time intervals conventional APF gel group showed statistically significant decrease according to the conventional NaF iontophoresis and control groups. There was no statistically difference between conventional NaF iontophoresis and control groups at this time intervals ( Table 3).

**Table 3:**
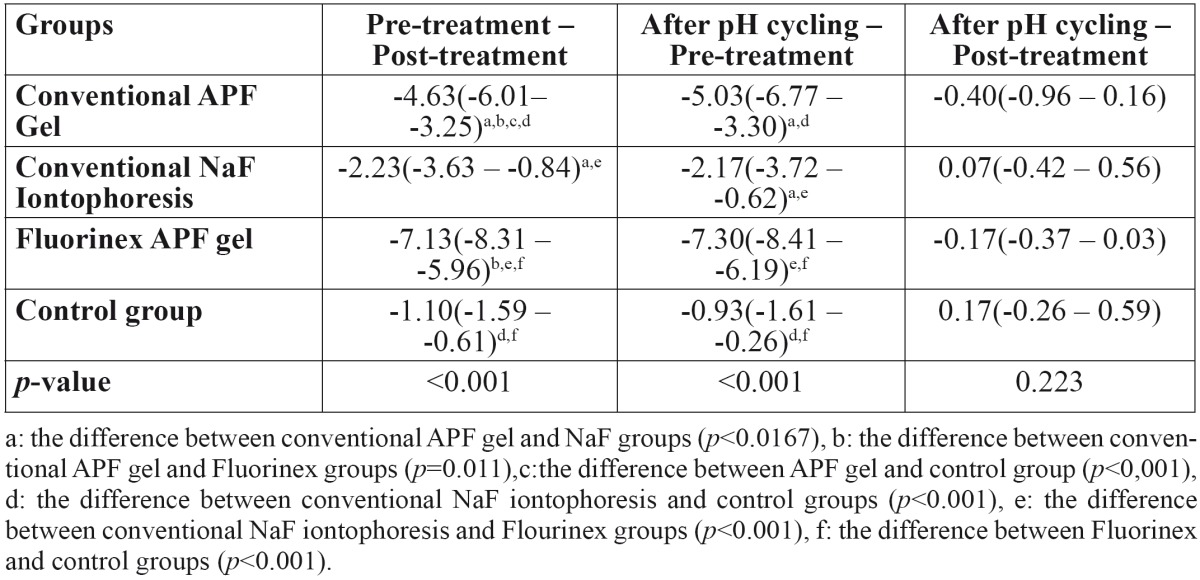
The distribution of the change in DIAGNOdent pen scores to the groups according to the measurement times.

The DIAGNOdent pen scores obtained pretreatment and after pH cycling showed that there was no statistically significant difference between Fluorinex and conventional APF gel groups [*p*=0.059]. But these groups showed statistically significant superiority than the others at this time intervals. After treatment and after pH cycling among the groups, no statistically significant difference was obtained from the DIAGNOdent pen measurements [*p*=0.223] ( Table 2). According to the measurement times, the alterations among the DIAGNOdent pen values showed no statistical significant difference between mesial and distal side of the teeth [*p*=0.078].

- Scanning Electron Micrographs

**Figure 1:**
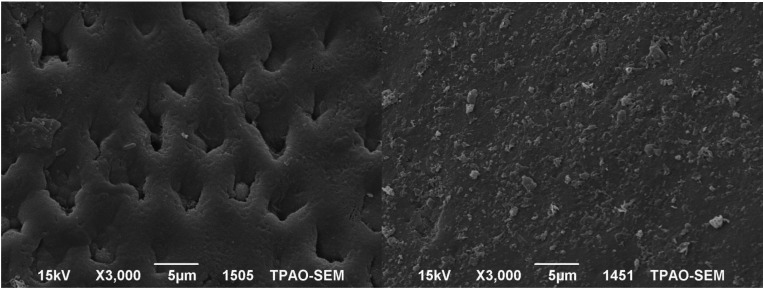
Left side: SEM images after demineralization. Right side: SEM images after conventional APF gel treatment.

**Figure 2:**
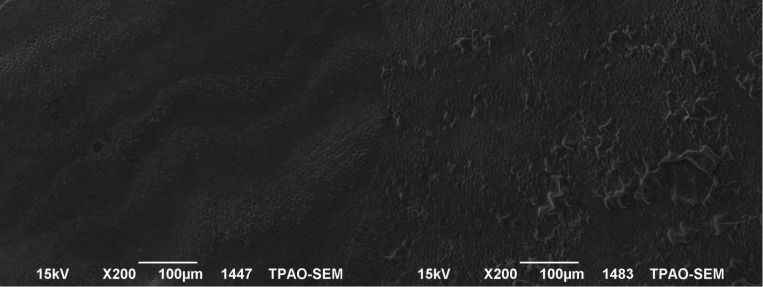
Left side: SEM images after demineralization, Right side: SEM images after conventional NaF iontophoresis.

**Figure 3:**
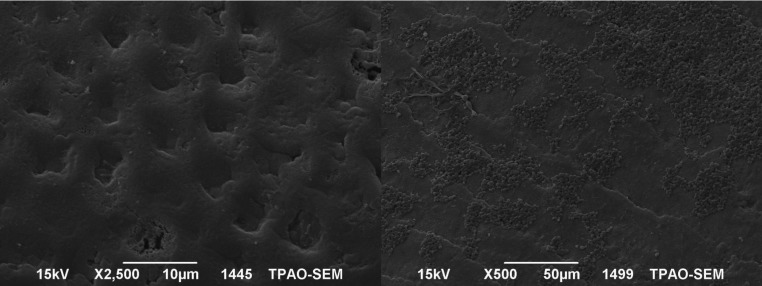
Left side: SEM images after demineralization. Right side: SEM images after APF gel treatment by Fluorinex.

**Figure 4:**
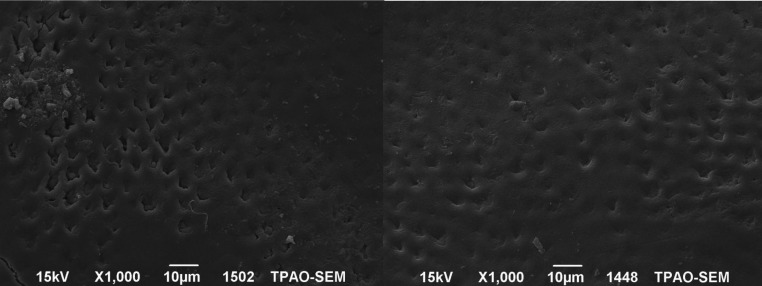
Left side: SEM images after demineralization. Right side: SEM images after placing in distilled water.

The SEM images of the mesial and the distal halves of the same tooth after demineralization and after re-mineralization are representative for each group are shown in figures 1-4. In figure 1 left side shows the enamel pores that were created after demineralization and in the right side the homogeneously precipitation of the amorphous CaF2 globules after conventional APF gel treatment could be observed. SEM images of an example from NaF iontophoresis group are shown in figure 2. Although applying NaF by iontophoresis the crater-like holes according the enamel prism exposure still be observed, but also in many areas precipitation of CaF2 could be seen. In Fluorinex group the characteristic honeycomb structure of demineralized enamel was clearly evident. After fluorid application by Fluorinex, it could be clearly seen that the surfaces are covered with CaF2 globules, which completely obscures the underlying prism structure and areas that clear globuler structure were not followed, a surface similar to that of healthy enamel was observed (Fig. 3). In figure 4 left side shows the SEM image after demineralization and right side is the SEM images after placing in distilled water. As hoping no therapeutic character of distilled water, images did not show any pronounced change.

## Discussion

The caries-inhibiting effect of fluoride was initially thought to be primarily due to incorporation of fluoride into the crystal lattice during development of the teeth. Today, it is suggested that post-eruptive interaction of fluoride with enamel is more important for caries protection. The caries preventive effects of fluoride are attributed to fluoride induced enhancement of remineralization, inhibition of demineralization, and reduction of dissolution after fluoride incorporation into the enamel crystallites. For caries prevention it seems to be essential to achieve high amounts of KOH-soluble fluoride on the enamel surface (15). However, some investigators showed that CaF2 on the enamel surface is easily dissolved within 24 h (3). To provide more fluoride retention by enamel and more time of oral fluoride release, practitioners have applied different fluoride agents in different concentrations by different clinical procedures, like by iontophoresis (16).

In the literature it is observed that specially demineralization and remineralization studies have been carried on in vitro situation. Hatibovic-kofman *et al.* (17) reported that *in vitro* studies is very important for obtaining specifying factors of therapeutic agents and to new agents enter the clinical applications. Based on this information this study was carried on *in vitro* conditions.

In *in vitro* studies usually bovine or human teeth are used. In this study human teeth were preferred, however the composition of the human teeth varies depending on sclerosis, genetic factors, fluoride intake and feeding before maturation and enviromental factors such as caries attacks. Aiming to minimize these factors, this study was carried out on unmatured premolars and impacted third molars, and comparisons were done in the halves of the same tooth. To investigate the demineralization and remineralization differences on the mesial and distal side of the same tooth, laser fluorescence measurement were recorded from two sides. However, according the measurement times, the alterations among the DIAGNOdent pen values showed no statistical significant difference between mesial and distal side of the same tooth [*p*=0.078]. This conclusion was similar as Rodrigues *et al.* (18) arrived from a previous study. Results showed numerical differences at different sides, but it meaned no statistical significancy. It is thought that to determine minimal mineralization alterations from mesial and distal sides, more sensitive analysis techniques is needed.

Laser fluorescence device is one of the most commonly used methodology in restorative dentistry, as it provides a simple, quantitative and comparable method of evaluating the performance of the various techniques (19). DIAGNOdent pen is a portable laser flourescence device which can detect the mineral loss in enamel before any cavitation occurs. In a recent study, Aljehani *et al.* (20) showed that the correlation between DIAGNOdent and visual examination was 0.63 and reported that DIAGNOdent was more objective and reproducible method than visual examination in detecting and long-term following up the incipient carious lesions. In this study incipient carious lesions were created by lactic acid gel technique as Takagi *et al.* (21) and detected by DIAGNOdent pen, the measurements showed the similar laser fluoresence values with the other studies which proved the occuring artificial lesions (22). Reis *et al.* (23) compared the performance of the DIAGNOdent in *in vitro* and *in vivo* studies and reported the accuracy in in vitro studies was higher. In this line, the *in vitro* situations of this study is the other factor that decreases the error rate in DIAGNOdent pen measurements. As well as the studies that suggest DIAGNOdent pen have to be used with visual examination, there are lots of studies that used the device alone (19) or with SEM (22) examination and reported the reliable results when the device used alone, like these studies in this study the results that were obtained by DIAGNOdent pen was supported with SEM images.

It is known that consantration, pH and application time of the topical agents are the effective factors on remineralization of enamel (24,25). When remineralization researchs carried out up to date is evaluated, there was no standardization about application time of fluoride agents is determined. Thus in the present study, as described in instructions’ manuals of the devices used, and shown in the similar remineralization researchs fluoride application time was set at 4 minutes.

According to the laser fluorescence scores, conventional APF gel and Fluorinex APF gel groups showed higher decreases in the scores than NaF iontophoresis groups. Several previous studies demonstrated that acidulated fluoride application is more effective than neutral fluoride application in remineralization. This fact can be explained by the fact that acid dissolves the surface of the enamel, and there can be formation of CaF2 due to calcium from the teeth as well as fluoride is likely to penetrate into the enamel structure (26,27).

On the other hand remineralization with the Fluorinex was superior to that of conventional APF gel. These results support the results of studies that showed further fluoride ion penetration and furher CaF2 precipitation by iontophoresis. Besides that, in the Fluorinex group the intensive and further CaF2 globules precipitation than the other groups could be observed by SEM analysis in this research.

Unpredictably conventional NaF group showed no statistically significant difference before and after the fluoride treatment [*p*>0.001] when control group showed statistically significant difference among the values obtained after demineralization and after distilled water application [*p*<0.001]. This can be a result of this study’s constituted as a mixed type study [region; mesial or distal, groups and time periods]. Before testing the significant differences, the interaction terms were analysed and the interaction results indicated that only group and time was found significant ( Table 1).

When the DIAGNOdent values obtained after demineralization and after pH cycling were compared, only in the APF gel and Fluorinex group there was a statistically significant decrease at values [*p*<0.001]. It can be assumed that a single dose application of APF by conventional technique or by Fluorinex created loosely bound fluoride and after 2 weeks it still plays an important role, due to its remineralizing capacity. As in other *in vivo* and *in situ* studies (28,29), Buchalla *et al.* (30) reported that a single dose application of highly concentrated fluoride agents could create KOH soluble fluoride and after 4 weeks decreased to minimum amount. It is likely that fluoride made available from the KOH soluble fluoride reservoir contributed to this remineralization. It is also most likely that fluoride released from the CaF2-like precipitates was incorporated into the gained mineral and accumulated structurally bound. This may explain the long-lasting efficacy of highly concentrated fluoride products applied only 2 or 3 times annually.

Results of this study indicate that APF gel application by Fluorinex ensures remineralization of incipient enamel lesions. But further study is needed for this application technique could be an alternative to conventional fluoride applications in clinics.
